# TRENDS IN MENTAL ILLNESS ACROSS ASSISTED LIVING SETTINGS: RESIDENT AND COMMUNITY FACTORS

**DOI:** 10.1093/geroni/igac059.2699

**Published:** 2022-12-20

**Authors:** Bryant Carlson, Sarah Dys, Ozcan Tunalilar, Paula Carder

**Affiliations:** Oregon Health & Science University - Portland State University School of Public Health, Portland, Oregon, United States; Portland State University, Portland, Oregon, United States; Portland State University, Portland, Oregon, United States; Portland State University, Portland, Oregon, United States

## Abstract

Although rates of serious mental illness (SMI), depression, and anxiety are becoming more common among assisted living (AL) and residential care (RC) residents, few studies have investigated resident and community-level factors to ensure that the settings and resources are adequate to meet residents’ mental health needs. Using a representative sample of Oregon AL/RC residents (n = 1,013), this study uses descriptive cross-sectional analysis to examine intrastate variation in the prevalence of SMI, depression, and anxiety related to: (1) resident-level characteristics (e.g., mental illness comorbidity, psychotropic medication use, and Medicaid status); (2) community-level characteristics (e.g., facility type and urban/rural geographic designation); and (3) health services utilization (e.g., emergency room visits and hospital admission). Results indicate that 12% of AL/RC residents had an SMI diagnosis, nearly half of whom also had depression and anxiety, and 80% were Medicaid eligible. One in six residents with SMI received at least three psychotropic medications in the last week. Residents with SMI were also more likely to live in RC facilities than AL facilities, in facilities with Medicaid contracts, and in urban rather than rural settings. Compared to residents without SMI, a larger share of residents with SMI utilized hospital emergency rooms (32% vs. 18%) and were admitted to the hospital overnight (15% vs. 9%). Findings underscore characteristics associated with potentially higher needs among AL/RC residents with SMI compared to their counterparts, with implications for the high quality provision of mental health services and quality of life and care in these settings.

